# How apoptotic β-cells direct immune response to tolerance or to autoimmune diabetes: a review

**DOI:** 10.1007/s10495-015-1090-8

**Published:** 2015-01-22

**Authors:** Marta Vives-Pi, Silvia Rodríguez-Fernández, Irma Pujol-Autonell

**Affiliations:** Immunology Department, Germans Trias i Pujol Research Institute, Universitat Autonoma de Barcelona, Carretera Canyet s/n, 08916 Badalona, Spain

**Keywords:** Apoptosis, Type 1 diabetes, Efferocytosis, β-Cells, Tolerance, Autoimmunity

## Abstract

Type 1 diabetes (T1D) is a metabolic disease that results from the autoimmune attack against insulin-producing β-cells in the pancreatic islets of Langerhans. Currently, there is no treatment to restore endogenous insulin secretion in patients with autoimmune diabetes. In the last years, the development of new therapies to induce long-term tolerance has been an important medical health challenge. Apoptosis is a physiological mechanism that contributes to the maintenance of immune tolerance. Apoptotic cells are a source of autoantigens that induce tolerance after their removal by antigen presenting cells (APCs) through a process called efferocytosis. Efferocytosis will not cause maturation in dendritic cells, one of the most powerful APCs, and this process could induce tolerance rather than autoimmunity. However, failure of this mechanism due to an increase in the rate of β-cells apoptosis and/or defects in efferocytosis results in activation of APCs, contributing to inflammation and to the loss of tolerance to self. In fact, T1D and other autoimmune diseases are associated to enhanced apoptosis of target cells and defective apoptotic cell clearance. Although further research is needed, the clinical relevance of immunotherapies based on apoptosis could prove to be very important, as it has translational potential in situations that require the reestablishment of immunological tolerance, such as autoimmune diseases. This review summarizes the effects of apoptosis of β-cells towards autoimmunity or tolerance and its application in the field of emerging immunotherapies.

## Introduction

Type 1 diabetes (T1D) is an autoimmune disease that permanently destroys the insulin-producing cells in the pancreatic islets as a consequence of T-lymphocyte mediated autoimmunity. There is a long asymptomatic period -prediabetic phase- characterized by the production of autoantibodies specific for islet autoantigens, including insulin. The inflammatory response in the islet microenvironment contributes to the induction and amplification of the immune attack against pancreatic β-cells and at later stages, to the stabilization and maintenance of insulitis [1], mainly composed by T and B cells, but also macrophages, dendritic cells (DCs), natural killer (NK) cells and natural killer T (NKT) cells. Moreover, TNF-α and IFN-γ produced by leukocytes in the islet microenvironment may induce nitric oxide (NO) secretion by β-cells, thus promoting their apoptosis [2]. In this scenario, proinflammatory cytokines and chemokines may contribute to suppress β-cell function and to induce apoptosis, and also to the recruitment of antigen presenting cells (APCs) into the pancreatic islets. These APCs can activate the specific immune response or act as regulatory cells to maintain tolerance. The removal of apoptotic β-cells is crucial in this step. If β-cells are not efficiently cleared in a short period of time, they will become necrotic, contributing to autoimmune attack amplification, through autoreactive lymphocytes activation. In addition, β-cells from T1D patients display a low but constant capacity of regeneration [3], constituting a chronic source of apoptotic β-cells that may contribute to the proposed model of relapsing-remitting T1D [4]. Here we review how apoptotic β-cell clearance has immunological consequences on disease pathogenesis and how the design of innovative immunotherapies may be inspired by apoptosis.

## Apoptosis is a crucial process in homeostasis

In the human body, tens of billions of cells continuously die as a consequence of cell turnover, cell damage, infection or immunological central tolerance. This physiological cell death is programmed, and the main mechanism is called apoptosis [5]. During apoptosis, the cell and nuclei condense and become fragmented without losing functional integrity of the nuclear envelope and cell membrane. Morphological characteristics of apoptosis are membrane blebbing, nuclear fragmentation and chromatin condensation. This physiological process eliminates unwanted cells, maintains homeostasis and confers advantages to the organism.

The small sealed cell fragments resulting from apoptosis are called apoptotic bodies. This event stands in contrast to necrosis, which is first marked by a loss of cell membrane integrity and a release of damage-associated molecules. The formation of apoptotic bodies is a mechanism for preventing leakage of potentially toxic or immunogenic cellular contents of dying cells. Despite the constant turnover of cells through apoptosis, apoptotic cells are rarely seen under physiological conditions because of a high rate of apoptotic cell clearance by phagocytes. This process is called efferocytosis, from *efferre*, which means ‘to take to the grave’ or ‘to bury’ in Latin. Efferocytosis takes place without inducing inflammation, with an anti-protease activity and growth-promoting effects to help in cell replacement.

## Apoptosis and efferocytosis modulate the immune response

The immune system is one of the most complex networks of the body. Its main role is to recognize and protect the organism from invading pathogens, neoplastic cells, and other dangerous signals, while maintaining tolerance to self. The first and immediate line of defense to protect the organism from infection is provided by the innate immune response, which triggers an inflammatory response and initiates the response of the adaptive immune system. The adaptive immune response can distinguish between self/nonself antigens, being highly specific to each particular pathogen or dangerous antigen. A key feature of the adaptive immune system is memory, which confers life-long immunity to previously encountered pathogens. In principle, the immune system of a subject does not become activated against its own tissues, a concept proposed as *horror autotoxicus* at the beginning of the twentieth century by Paul Ehrlich [6]. However, the complex immunological network may fail in certain individuals or life stages, thus allowing the immune system to attack self-components of the body. This disorder is called autoimmunity, and can be demonstrated by the presence of autoantibodies and autoreactive T lymphocytes [7], capable of transferring the autoimmune reaction [8]. Autoimmunity is the cause of a broad spectrum of human illnesses, known as autoimmune diseases.

Dying cells talk to the immune system and alert the immune system if necessary [5]. If cell death is caused by a danger-trauma, cancer, infectious disease-, defense and repair mechanisms are mobilized in the host. However, if cell death is part of normal physiological processes, the immune system takes advantage of the cell removal to inhibit immune responses and to maintain tolerance to self, as demonstrated in experimental models [9, 10]. Whereas necrotic cells alert the immune system to respond, apoptotic cells initially maintain membrane integrity and, if they are rapidly cleared by phagocytes, these cells do not release danger signals and the immune system is not stimulated [11]. Therefore, efferocytosis promotes immune tolerance to autoantigens in the absence of inflammation [12], by keeping an ‘immunologically silent’ microenvironment [13].

Recent studies provide new findings into the process, including how APCs process apoptotic cells without inducing inflammation and maintaining cellular homeostasis [14]. Many receptors, adaptors and chemotactic molecules are involved in prompt apoptotic cell clearance [15]. Over the last few years, new insights into the engulfment process of apoptotic cells by phagocytes have been reported [5, 16]. In vivo cell clearance is performed through four steps: firstly, the sensing of the corpses is done by ‘find me’ signals released by apoptotic cells, such as chemokines (CX_3_CL1 [17]), adhesion molecules (intercellular adhesion molecule 3 (ICAM-3) [18]) and nucleotides (ATP and UTP [19]), among others. These signals are recognized by receptors in the membrane of phagocytes and induce phagocyte migration toward the apoptotic cell. Also, ‘stay away’ signals have been identified in order to maintain an anti-inflammatory microenvironment. In this sense, lactoferrin proteins released by apoptotic cells inhibit neutrophil recruitment [20]. Secondly, ‘eat me’ signals exposed on the surface of apoptotic cells are recognized by phagocyte receptors. One of the main ‘eat-me’ signals is phosphatidylserine (PS), translocated to the outer leaflet of the lipid bilayer in apoptotic cells. Many receptors that recognize PS on apoptotic cells have been described on the surface of phagocyte cells, such as members of the T cell immunoglobulin mucin domain (TIM) protein family including TIM-1 and TIM-4 [21, 22], the Stabilin-2 [23], the receptor for advanced glycation end products (RAGE) [24] and the brain-specific angiogenesis inhibitor 1 (BAI1) [25]. PS may also be recognized indirectly by bridging molecules, such as Gas6 and protein S through the TAM family of receptors (Tyro-3, Axl, and Mer) [26]. Other membrane molecules have also been described to bind apoptotic cells, such as CD36, CD14, CD68 and αVβ3 integrin [27], among others. In addition to ‘eat me’ signals, ‘don’t eat me’ signals, expressed on the surface of living cells, such as CD47, help phagocytes to distinguish between alive and dead cells [28]. Thirdly, signaling pathways regulate cytoskeletal rearrangement for engulfment, and finally, signaling events within the phagocytes regulate the processing of apoptotic cell autoantigens to induce tolerance to self in an immunologically silent microenvironment [29].

After efferocytosis, anti-inflammatory mediators are produced by the APCs—mainly DCs—whereas the release of inflammatory cytokines is inhibited by avoiding DCs maturation. DCs are the most professional APCs and determine immunogenicity or tolerance. DCs play a basic role in the initiation of the immune response by presenting antigenic peptides when activated, but in the absence of inflammation, immature DCs (iDCs) are essential to maintain tolerance to self. The capture of apoptotic cells by iDCs does not cause maturation and maintains peripheral tolerance [13, 30]. Moreover, a subset of DCs constantly uptake apoptotic cells and deliver tolerogenic signals to self in the lymph node [31]. Therefore, the uptake of apoptotic cells—which has been proposed to suppress DC immunogenicity—is a physiological mechanism involved in tolerogenic DCs (tolDCs) generation (Table 1). TolDCs are effector DCs capable of inducing peripheral tolerance. It has been shown that tolDCs loaded with apoptotic cells express low levels of MHC class II molecules and costimulatory molecules CD40, CD80 and CD86, even after the exposure to some proinflammatory stimulus [32, 33], secrete low amounts of proinflammatory cytokines IL-1α, IL-1β and TNF-α [34] and decrease their ability to stimulate T cell proliferation [35]. In addition, efferocytosis may induce DCs to secrete immunosuppressive mediators, such as TGF-β, which triggers the differentiation of classical regulatory T lymphocytes, Foxp3^+^ Tregs [36, 37]. In this sense, the secretion of IL-10, an anti-inflammatory cytokine, has been found upregulated in LPS-activated DCs exposed to early apoptotic cells. Those DCs become impaired to maturate and fail to activate T cells [38]. Other authors reported that apoptotic cells could turn DCs to inhibitory DCs, impairing CD4^+^ T cell proliferation via nitric oxide (NO) production [39, 40]. Efferocytosis may also promote suppressive effects on DCs through prostaglandin E_2_ (PGE_2_) production [41] or via interferon-γ-induced indoleamine 2,3-dioxygenase (IDO) [42]. It has also been reported that the tolerogenic effect acquired by DCs after efferocytosis results in tolerance induction in vivo. For example, the uptake of apoptotic cells by viable DCs is able to suppress their maturation and promote tolerance through the induction of Tregs by the secretion of TGF-β [43] or the expression of programmed death-ligand 1 (PD-L1) [44]. In the context of transplant immunology, the administration of donor apoptotic cells prolonged cardiac allograft survival in mice [45] and improved chronic allograft vasculopathy [46] through the interaction with recipient DCs. In addition, recipient DCs loaded with donor-derived apoptotic cells restrained allorecognition in cardiac allograft transplant [47].

**Table 1:** Characteristics of tolerogenic DCs generated by efferocytosis

Characteristics	Effect	DCs source (mice/human)	Bibliography
Low CD40, CD80, CD86, MHC class II expression	Incomplete antigen presentation	BMDCs (C57BL/6, BALB/c, NOD mice) and splenic DCs (BALB/c mice)/MoDCs (healthy donors)	[32, 33, 35, 44, 71]
Low IL-1α, IL-1β, IL-6 and TNF-α profile	Non-inflammatory microenvironment	BMDCs (C57BL/6, C57BL/10, NOD mice)	[32, 34, 71]
Increased PGE_2_ secretion	Suppression of T cell proliferation	BMDCs (NOD mice)	[41]
Increased TGF-β secretion	Induction of Tregs	BMDCs (C57BL/6, BALB/c mice)	[37, 36, 43]
Increased NO production	Suppression of T cell proliferation	BMDCs (C57BL/6, BALB/c mice)	[39, 40]
Increased IDO expression	Suppression of T cell proliferation	MoDCs (healthy donors)	[42]
Increased IL-10 secretion	Anti-inflammatory	MoDCs (healthy donors)	[38, 93]
Increased PD-L1 expression	Induction of Tregs	Splenic DCs (BALB/c mice)	[44]
Stability	LPS maturation resistance	BMDCs (C57BL/6, BALB/c, NOD mice)/MoDCs (healthy donors)	[32, 33, 36, 37, 38, 41]

*DCs* dendritic cells, *BMDCs* bone marrow-derived dendritic cells, *NOD* non obese diabetic, *MoDCs* monocyte-derived dendritic cells, *TNF* tumor necrosis factor, *PGE*_*2*_ prostaglandin E_2_, *TGF* transforming growth factor, *NO* nitric oxygen, *IDO* indoleamine 2,3-dioxygenase, *PD-L1* programmed death-ligand 1, *LPS* lipopolysaccharide

In apparent contradiction to these data, apoptotic cells induce macrophages or DCs activation in some cases. It is well known that DCs that engulfed infected or tumor apoptotic cells can induce physiological anti-viral and anti-tumor antigen specific immunity [48]. This fact could be explained by differences on the antigenic content of harmless or dangerous apoptotic cells. In fact, several aspects of the apoptotic process are basic for the subsequent immune modulation [16]: (1) The ‘quality’ of apoptotic cells, which includes the cell type, the cause of cell death and the activation status of the dying cells. It determines which type of ‘eat me’ signals is being exposed to the phagocytes [15]. (2) The ratio of apoptotic cells, which can determine the magnitude of the resulting immune response. (3) The apoptotic cell microenvironment that determines which phagocyte is in charge to mediate clearance and regulate the immune response. (4) The timing of cell death and duration of apoptotic cell-derived signals. Therefore, the specific conditions of apoptosis define if apoptotic cells promote immunity or induce tolerance.

Apoptosis can be pathological, due to either too much loss of cells with little regenerative capacity, by too little apoptotic cell removal or by ineffective apoptotic cell digestion. These processes are altered in many diseases, including autoimmune diseases [11]. If apoptotic cells are not removed or digested within a certain time, they become late apoptotic cells -called secondary necrotic cells- that have lost the integrity of the membrane, favoring the release of intracellular content. In this case, these cells can promote inflammation and autoimmunity, similar to necrotic cells. Defects in clearance are believed to have an important effect on autoimmune diseases, cardiovascular diseases, neurological diseases, pulmonary diseases, infection and cancer [49]. Moreover, it has been described that accumulation of DNA from apoptotic cells -due to an excess of apoptosis and/or defective degradation mechanisms in the cytoplasm of phagocytes- could induce excessive type I IFN production [50]. The production of these cytokines could be a triggering factor in the process leading to autoimmune diseases in general and to T1D in particular [51].

## Type 1 diabetes: apoptosis of β-cells and implications for the disease

T1D is a metabolic disease that results from the autoimmune attack against insulin-producing β-cells in the islets of Langerhans of the pancreas. The islets, taken together, can be thought of as a single organ occupying 1 % of the pancreas weight. Each islet contains on average a total of 1,000 cells [52] that secrete hormones related to the glucose homeostasis. Among these, 65–80 % are insulin-producing β-cells. T1D etiology is unidentified, but it is known that the disease appears in genetically susceptible individuals with impaired immune regulation after environmental triggers [53]. The onset of the disease is preceded by a long asymptomatic period named prediabetes. During this prediabetic stage, islet reactive autoantibodies arise in the sera and autoreactive T cells start to destroy β-cells, resulting in a progressive loss in insulin secretory function. Clinical onset of T1D occurs when 80–90 % of the β-cells have been destroyed.

Apoptosis of β-cells has been demonstrated to be involved in autoimmune T1D and type 2 diabetes (T2D), as in the loss of insulin producing cells after islet transplantation [54]. In T2D abnormal levels of metabolic factors contribute to the apoptosis after β-cell failure [55]. The decrease of β-cell mass after islet transplantation can be due to several factors, including apoptosis triggered by the stress related to enzymatic islet digestion and purification [56], and allogeneic graft rejection. T1D is associated to enhanced β-cells apoptosis and defective clearance. In this sense, much of our knowledge of the role of apoptosis in T1D comes from the non obese diabetic (NOD) mouse, a spontaneous murine model that closely resembles human T1D [57]. In health conditions, a peak of physiological β-cell death has been described in mice 2 weeks after birth [58]. Interestingly, a similar peak of β-cell death has been described in humans, peaking at the perinatal period, when the process of islet remodeling has been observed [59]. This wave of physiological β-cell death, which takes place in all individuals, seems to be very important in the initiation of T1D in genetically predisposed subjects [60]. In addition to these waves of β-cell death, several factors may promote β-cell apoptosis such as cytokines, glucose, hypoxia and different forms of stress [61]. Prolonged exposure of pancreatic β-cells to proinflammatory cytokines induces the expression of miRNAs that contribute to β-cell dysfunction and apoptosis during the early asymptomatic stages of autoimmunity [62]. Therefore, the wave of physiological β-cell death is crucial in the initiation of T1D, due to either too much loss of cells with little regenerative capacity or by too little apoptotic cell removal [60]. If these dying β-cells are not efficiently removed, they release danger signals that may activate DCs promoting insulitis progression and diabetes onset in NOD mice [63].

It has been proposed that the initiation of the disease occurs in the islets of the pancreas, where under undefined pathogenic conditions, β-cell antigens are released. The alterations in the pancreatic islets in humans at T1D onset have been described in a small number of observations from autopsies [64, 65] and few pancreatic biopsies of T1D patients [66]. Interestingly, increased Fas expression was identified in β-cells of inflamed human islets, which can induce apoptosis after binding to its receptor [67]. In this sense, differential expression of genes related to apoptosis has been reported in human pancreases from T1D patients [65, 68]. These changes sensitize β-cells to proapoptotic signals (e.g., cytokines), modulating apoptosis in preclinical T1D autoimmunity. By contrast, islets from T1D patients also showed down-regulation of pro-apoptotic genes: *MLLT11*, *PRUNE2* and *NLRP1*, probably reflecting an attempt to arrest β-cell apoptosis. Therefore, the progression towards T1D derives from the imbalance between increased β-cell death and deficient maintenance of β-cell mass.

## Efferocytosis of β-cells is critical in autoimmune diabetes

**Fig. 1:**
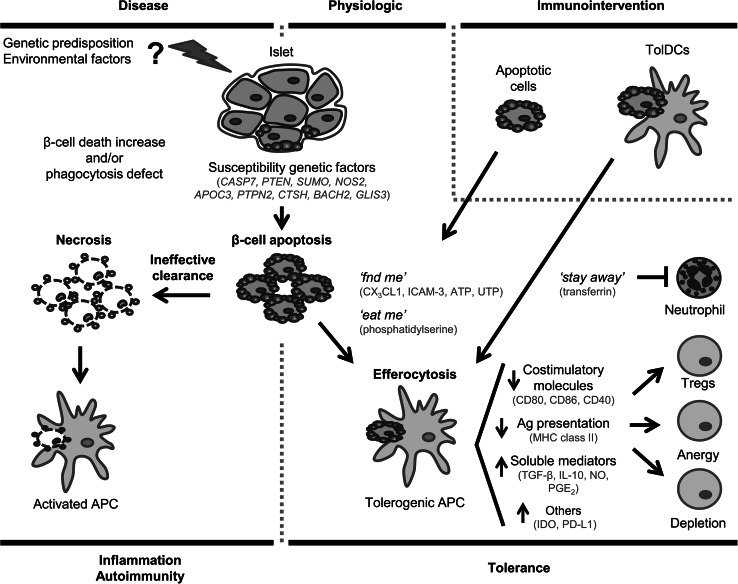
Schematic representation of β-cells apoptosis and efferocytosis in type 1 diabetes. β-cells that undergo apoptosis are recognized and phagocytosed by APCs, leading to physiologic tolerance induction. However, an increase in the rate of β-cells apoptosis rate and/or defects in efferocytosis result in the activation of APCs, contributing to inflammation, to the lack of tolerance to self and to autoimmune disease. Strategies of immunointervention based on apoptosis, such as apoptotic cell administration or tolDCs-pulsed with apoptotic β-cells- could help to the reestablishment of immunological tolerance to β-cells autoantigens, lost in type 1 diabetes

Apoptotic β-cells are the most important source of autoantigens in T1D. A high rate of β-cell apoptosis or defects in the removal of apoptotic cells in pre-diabetes contribute to autoimmunity [60], because these cells turn into late apoptotic bodies and secondary necrotic cells, favoring inflammation, insulitis and autoimmunity (Fig. 1).

In the NOD mouse model, a deficiency in the clearance of apoptotic cells by macrophages has been described [69]. The accumulation of apoptotic cells results in necrosis, favoring inflammation and recruitment of macrophages, DCs, and T cells to the islets, thus exacerbating autoimmune damage. It has been reported that a low but constant capacity of regeneration of β-cells exists in longstanding T1D patients [3]. This suggests a relapsing-remitting nature of the disease [4]. In this sense, β-cell regeneration could have a dual role in T1D, trying to recover β-cell mass but promoting the autoimmune process through a defective efferocytosis, leading to diabetes in a vicious cycle.

As it has been previously mentioned, DCs play a dual role in the immune system. Therefore, how can iDCs induce tolerance to autoantigens after apoptotic β-cells efferocytosis? The low levels of costimulatory molecules in the DC (CD80, CD86), the possibility of a subset of DCs specialized in tolerance induction, the anti-maturation cytokine profile, and the expansion of regulatory T cells could be key factors in maintaining tolerance to autoantigens from apoptotic cells generated during the normal tissue turnover [70]. Our group has shown that after the uptake of apoptotic β-cells, DCs displayed a molecular switch to a tolerogenic phenotype impairing the progress of insulitis in the NOD mouse, resulting in the prevention of the disease [71]. DCs loaded with apoptotic β-cells diminish the expression of costimulatory molecules CD40 and CD86, secrete low amounts of proinflammatory cytokines and acquire impaired ability to stimulate autologous T cell proliferation, even after proinflammatory stimuli, thus demonstrating their stability [41]. The initial events leading to DC activation in T1D are still unknown, but evidence points to a complex interaction between cytokines, chemokines, costimulatory molecules, inflammatory mediators, β-cell cytotoxicity and regeneration, and attempts of immunoregulation in the islet microenvironment [72]. The β-cell apoptosis/clearance rate would be decisive in DC activation and insulitis progression.

The suppressive function of DCs acquired after efferocytosis is a key feature in the maintenance of tolerance to self. After uptake of apoptotic cells, DCs suppress T cell proliferation induced by mature DC, thus indicating the existence of an active suppressor mechanism involving PGE_2_ production [73]. PGE_2_ is a lipid mediator produced by the liberation of arachidonic acid from cell membrane phospholipids that has been generally designed to modulate inflammation, promoting local vasodilatation and local attraction and activation of innate immune cells at early stages of inflammation. However, PGE_2_ also has a suppressive function that limits inflammation [74] to protect against immunity to self-antigens. In agreement with this, it has been proposed that PGE_2_ is released during tissue injury and inflammation to protect against immunity to self antigens [75]. In context of T1D, efferocytosis promotes suppressive effects on DCs through PGE_2_ production [41]. Moreover, PGE_2_ levels were found significantly higher in T1D patients [76], maybe as a response to the inflammation in an immunoregulatory attempt.

Efficient efferocytosis by DCs and other APCs involves ‘stay away’ signals to inhibit neutrophil recruitment. One of these signals is transferrin, an iron binding protein. In fact, transferrin administration protects against disease in NOD mice [77]. In humans, a decrease in transferrin levels was described in the sera of newly diagnosed T1D patients. Moreover, neutrophil recruitment observed in the pancreas of donors with T1D [78] is controlled by the overexpression of neutrophil attracting chemokines (CXCL1, CXCL2) [65, 79]. The cross talk between neutrophils-recruited in a defective process of apoptotic cell clearance-, B-1a and plasmacytoid DCs leads to autoimmune diabetes in NOD mice [80]. Therefore, we hypothesize that defects in ‘stay away’ signals in early β-cells efferocytosis may contribute to the loss of tolerance.

## Genetics of β-cells apoptosis: additional genetic susceptibility factors in type 1 diabetes?

There is a strong genetic susceptibility component in T1D: 50 % of the genetic predisposition is due to polymorphisms in the HLA class II region (named IDDM1) in chromosome 6. Genome-wide association studies (GWAS) have identified more than 50 loci associated with genetic risk of type T1D. In addition to HLA genes, other *loci* associated to T1D are the human insulin promoter that controls the expression of insulin (IDDM2) and immune-related genes such as *PTPN22*, *CTLA*-*4*, *IL2RA*, *IFIH1*, among others (named IDDM3 to IDDM18) [81]. Most of these genes have immunological functions, whereas some have metabolic functions, but the exact mechanisms by which these loci confer susceptibility to T1D remain elusive. New T1D susceptibility regions can be continuously identified through association studies (T1Dbase, www.t1dbase.org).

Because apoptosis is a key factor in T1D, there might be a link between genetic alterations or polymorphisms in the apoptosis machinery and the pathogenesis of the disease. Although no genetic defects in apoptosis pathways have been specifically identified in T1D, functional assays demonstrate increased apoptosis attributable to the multiple pathways of this type of cell death and several susceptibility genes have been reported. In this sense, genes linked to T1D are caspase 7 (*CASP7*), phosphatase and tensin homolog (*PTEN*), small ubiquitin-like modifier (*SUMO*), nitric oxide synthase-2 (*NOS2*) and apolipoprotein CIII (*APOC3*) genes [82]. These gene products are important for apoptosis induction (*CASP7* and *PTEN*), and regulation (*SUMO*), for NO production and apoptosis of β-cells (*NOS2*), and for promoting apoptosis through increasing calcium concentration (*APOC3*). Moreover, recent studies indicate that important genetically regulated pathways in β-cells involve genes related to β-cell susceptibility to apoptosis. One of these genes, protein tyrosine phosphatase, non-receptor type 2 (*PTPN2*), regulates cytokine-induced apoptosis and may thereby contribute to the pathogenesis of T1D [83, 84]. Another candidate is the cathepsin H (*CTSH*) gene that exerts its antiapoptotic effects through reduced expression of proapoptotic factors Bim, DP5 and c-Myc [85]. Also, basic leucine zipper transcription factor 2 (*BACH2*) has been associated to genetic risk of developing autoimmune diabetes [86]. This gene regulates proinflammatory cytokine induced apoptosis in β-cells by cross talking with *PTPN2*. Besides, gli-similar 3 (*GLIS3*) may favor β-cell apoptosis by modulating the alternative splicing of the proapoptotic BH3-only protein Bim and exacerbating the formation of the most proapoptotic variant Bim_S_ [87, 88].

In the context of islet autoimmunity, there is a cross talk between inflammation, adaptive immunity and β-cells, influenced by genetic polymorphisms that affect β-cells gene expression and amplify apoptosis or diminish anti-apoptotic signals [89]. A possibility is that subjects carrying these candidate genes can display enhanced β-cell apoptosis and consequently, defective apoptotic cell clearance or secondary necrosis. These defects are detrimental for apoptosis-induced self-tolerance, resulting in the activation of APCs and the presentation of autoantigens to the immune system. This may permit the loss of tolerance and the activation of autoreactive T lymphocytes thus potentially amplifying β-cell death and insulitis and contributing to autoimmunity. The correlations between genotypes and phenotypes suggest an association between polymorphisms, tendency to β-cell apoptosis and susceptibility to T1D, but further studies are required to confirm that these genes play pathogenic roles in autoimmune diabetes. Understanding the genes responsible for β-cell apoptosis -but also to neogenesis and regeneration- may lead to the understanding of the imbalanced apoptosis rate in prediabetes and during hyperglycemia and may help to the development of new therapies to treat and prevent the disease.

## Use of apoptotic cells in emerging immunotherapies for type 1 diabetes

During the last years, considerable efforts have been made in generating new immunotherapies for T1D. However, no therapy has yet been able to prevent or cure human T1D. Ideally, a T1D immunotherapy should inhibit autoimmune attack against β-cells, avoiding systemic side effects and allowing tissue regeneration.

Apoptotic cells display inherent immunomodulatory properties useful to design antigen-specific immunotherapies in experimental models of T1D. Evidence linking apoptosis and tolerance has been found in several studies where the administration of autologous apoptotic cells led to tolerance induction in models of inflammation, such as inflammatory arthritis [90], induced lung inflammation [91, 92] or sepsis [40]. When engulfed by APCs, such as DCs, apoptotic cells contribute to the maintenance of self-tolerance through presentation of autoantigens in an active suppressive process that constitutes a silencer event [93]. The physiological uptake of apoptotic β-cells by DCs induces a preventive effect in experimental T1D [94]. In fact, transfusion of apoptotic islet cells decreased diabetes incidence in NOD mice [95], supporting the suppressor role of apoptotic cells. The administration of islet apoptotic cells loaded into tolDCs is another strategy to restore tolerance to insulin producing cells that prevents T1D in NOD mice [71]. Apoptotic cell engulfment promotes molecular and functional changes in DCs towards a tolerogenic phenotype in the context of T1D [41]. Gene expression profile of DCs from NOD mice that capture islet cell apoptotic bodies showed a downregulation of genes involved in antigen presentation and differential expression of cytokines, chemokines, natural immunity and immunoregulation genes, together with the presence of specific transcripts for T1D autoantigens. Autologous T cell proliferation decreases after the capture of apoptotic cells by DCs, together with a fall of proinflammatory, Th1 and Th17 cytokine secretion, even after a proinflammatory stimulus. Suppressive function of DCs acquired after efferocytosis of β-cells in NOD mice is mediated, at least in part, by PGE_2_, as previously mentioned. In summary, DCs can be programmed to induce specific immune tolerance to β-cells using apoptotic cells, constituting a rational strategy for a variety of autoimmune conditions. Several authors have generated tolerogenic DCs capable of modulating autoimmunity in experimental T1D [96]. In the clinical setting, a phase I trial has assessed tolerogenic DCs safety in T1D patients with good results [97]. However, the process of obtaining of apoptotic bodies from β-cells is very complex and an alternative source is required.

We are fully conscious that the NOD mouse strain has some immunological differences compared to human T1D. However, experimental models are relevant for dissecting tolerance mechanisms and to test experimental immunotherapies. Obviously, caution is needed when translating results from animal models to a clinical setting. First, diabetic patients do not share all the immune defects existing in the NOD mouse. Second, human β-cells are less able to regenerate than rodent β-cells. Third, NOD mice, unlike humans, are genetically identical. An important pre-clinical step will be the assessment of the in vitro effects of the apoptosis-based immunotherapies in human cells DCs from diabetic patients. In any case, efforts should be put into guiding translational research to successful clinical therapy for T1D.

## Conclusions

The efficient clearance of apoptotic β-cells by APCs in the islet microenvironment is crucial for the maintenance of tolerance to self. The interaction between the most professional APCs—DCs—and apoptotic β-cells is complex but it is well accepted that it contributes to the maintenance of self-tolerance. The breakdown of tolerance to β-cells is the cause of T1D, an autoimmune disease mediated by pathogenic T cells. As outlined in this review, an increase in the number of apoptotic β-cells—physiological or induced by stress in the islets—together with an inefficient efferocytosis further facilitate the loss of tolerance to T1D autoantigens and subsequent specific attack to the β-cells. Detailed knowledge of the mechanisms involved in an efficient efferocytosis may contribute to the development of innovative immunotherapies aimed to recover tolerance to self in autoimmune diseases, such as T1D.

## References

[1] Eizirik DL, Colli ML, Ortis F (2009). The role of inflammation in insulitis and beta-cell loss in type 1 diabetes. Nat Rev Endocrinol.

[2] Cnop M, Welsh N, Jonas JC (2005). Mechanisms of pancreatic beta-cell death in type 1 and type 2 diabetes: many differences, few similarities. Diabetes.

[3] Meier JJ, Bhushan A, Butler AE, Rizza RA, Butler PC (2005). Sustained beta cell apoptosis in patients with long-standing type 1 diabetes: indirect evidence for islet regeneration?. Diabetologia.

[4] von Herrath M, Sanda S, Herold K (2007). Type 1 diabetes as a relapsing-remitting disease?. Nat Rev Immunol.

[5] Kono H, Rock KL (2008). How dying cells alert the immune system to danger. Nat Rev Immunol.

[6] del Guercio P (1993). The self and the nonself: immunorecognition and immunologic functions. Immunol Res.

[7] Rose NR, Bona C (1993). Defining criteria for autoimmune diseases (Witebsky’s postulates revisited). Immunol Today.

[8] Lampeter EF, McCann SR, Kolb H (1998). Transfer of diabetes type 1 by bone-marrow transplantation. Lancet.

[9] Rossi AG, Sawatzky DA, Walker A (2006). Cyclin-dependent kinase inhibitors enhance the resolution of inflammation by promoting inflammatory cell apoptosis. Nat Med.

[10] McGrath EE, Marriott HM, Lawrie A (2011). TNF-related apoptosis-inducing ligand (TRAIL) regulates inflammatory neutrophil apoptosis and enhances resolution of inflammation. J Leukoc Biol.

[11] Nagata S, Hanayama R, Kawane K (2010). Autoimmunity and the clearance of dead cells. Cell.

[12] Erwig LP, Henson PM (2007). Immunological consequences of apoptotic cell phagocytosis. Am J Pathol.

[13] Steinman RM, Turley S, Mellman I, Inaba K (2000). The induction of tolerance by dendritic cells that have captured apoptotic cells. J Exp Med.

[14] Ravichandran KS, Lorenz U (2007). Engulfment of apoptotic cells: signals for a good meal. Nat Rev Immunol.

[15] Krysko DV, D’Herde K, Vandenabeele P (2006). Clearance of apoptotic and necrotic cells and its immunological consequences. Apoptosis.

[16] Poon IK, Lucas CD, Rossi AG, Ravichandran KS (2014). Apoptotic cell clearance: basic biology and therapeutic potential. Nat Rev Immunol.

[17] Truman LA, Ford CA, Pasikowska M (2008). CX3CL1/fractalkine is released from apoptotic lymphocytes to stimulate macrophage chemotaxis. Blood.

[18] Torr EE, Gardner DH, Thomas L (2012). Apoptotic cell-derived ICAM-3 promotes both macrophage chemoattraction to and tethering of apoptotic cells. Cell Death Differ.

[19] Elliott MR, Chekeni FB, Trampont PC (2009). Nucleotides released by apoptotic cells act as a find-me signal to promote phagocytic clearance. Nature.

[20] Bournazou I, Pound JD, Duffin R (2009). Apoptotic human cells inhibit migration of granulocytes via release of lactoferrin. J Clin Invest.

[21] Kobayashi N, Karisola P, Pena-Cruz V (2007). TIM-1 and TIM-4 glycoproteins bind phosphatidylserine and mediate uptake of apoptotic cells. Immunity.

[22] Miyanishi M, Tada K, Koike M (2007). Identification of Tim4 as a phosphatidylserine receptor. Nature.

[23] Park SY, Jung MY, Kim HJ (2008). Rapid cell corpse clearance by stabilin-2, a membrane phosphatidylserine receptor. Cell Death Differ.

[24] He M, Kubo H, Morimoto K (2011). Receptor for advanced glycation end products binds to phosphatidylserine and assists in the clearance of apoptotic cells. EMBO Rep.

[25] Park D, Tosello-Trampont AC, Elliott MR (2007). BAI1 is an engulfment receptor for apoptotic cells upstream of the ELMO/Dock180/Rac module. Nature.

[26] Nakano T, Ishimoto Y, Kishino J (1997). Cell adhesion to phosphatidylserine mediated by a product of growth arrest-specific gene 6. J Biol Chem.

[27] Fadok VA, Bratton DL, Henson PM (2001). Phagocyte receptors for apoptotic cells: recognition, uptake, and consequences. J Clin Invest.

[28] Gardai SJ, McPhillips KA, Frasch SC (2005). Cell-surface calreticulin initiates clearance of viable or apoptotic cells through trans-activation of LRP on the phagocyte. Cell.

[29] Hochreiter-Hufford A, Ravichandran KS (2013). Clearing the dead: apoptotic cell sensing, recognition, engulfment, and digestion. Cold Spring Harb Perspect Biol.

[30] Morelli AE (2006). The immune regulatory effect of apoptotic cells and exosomes on dendritic cells: its impact on transplantation. Am J Transplant.

[31] Huang FP, Platt N, Wykes M (2000). A discrete subpopulation of dendritic cells transports apoptotic intestinal epithelial cells to T cell areas of mesenteric lymph nodes. J Exp Med.

[32] Takahashi M, Kobayashi Y (2003). Cytokine production in association with phagocytosis of apoptotic cells by immature dendritic cells. Cell Immunol.

[33] Verbovetski I, Bychkov H, Trahtemberg U (2002). Opsonization of apoptotic cells by autologous iC3b facilitates clearance by immature dendritic cells, down-regulates DR and CD86, and up-regulates CC chemokine receptor 7. J Exp Med.

[34] Morelli AE, Larregina AT, Shufesky WJ (2003). Internalization of circulating apoptotic cells by splenic marginal zone dendritic cells: dependence on complement receptors and effect on cytokine production. Blood.

[35] Stuart LM, Lucas M, Simpson C (2002). Inhibitory effects of apoptotic cell ingestion upon endotoxin-driven myeloid dendritic cell maturation. J Immunol.

[36] da Costa TB, Sardinha LR, Larocca R, Peron JP, Rizzo LV (2011). Allogeneic apoptotic thymocyte-stimulated dendritic cells expand functional regulatory T cells. Immunology.

[37] Kushwah R, Wu J, Oliver JR (2010). Uptake of apoptotic DC converts immature DC into tolerogenic DC that induce differentiation of Foxp3 + Treg. Eur J Immunol.

[38] Urban BC, Willcox N, Roberts DJ (2001). A role for CD36 in the regulation of dendritic cell function. Proc Natl Acad Sci USA.

[39] Zhong K, Song W, Wang Q (2012). Murine myeloid dendritic cells that phagocytose apoptotic T cells inhibit the immune response via NO. PLoS One.

[40] Ren G, Su J, Zhao X (2008). Apoptotic cells induce immunosuppression through dendritic cells: critical roles of IFN-gamma and nitric oxide. J Immunol.

[41] Pujol-Autonell I, Ampudia RM, Planas R (2013). Efferocytosis promotes suppressive effects on dendritic cells through prostaglandin E2 production in the context of autoimmunity. PLoS One.

[42] Williams CA, Harry RA, McLeod JD (2008). Apoptotic cells induce dendritic cell-mediated suppression via interferon-gamma-induced IDO. Immunology.

[43] Kushwah R, Oliver JR, Zhang J, Siminovitch KA, Hu J (2009). Apoptotic dendritic cells induce tolerance in mice through suppression of dendritic cell maturation and induction of antigen-specific regulatory T cells. J Immunol.

[44] Wu C, Zhang Y, Jiang Y (2013). Apoptotic cell administration enhances pancreatic islet engraftment by induction of regulatory T cells and tolerogenic dendritic cells. Cell Mol Immunol.

[45] Wang Z, Larregina AT, Shufesky WJ (2006). Use of the inhibitory effect of apoptotic cells on dendritic cells for graft survival via T-cell deletion and regulatory T cells. Am J Transplant.

[46] Wang Z, Shufesky WJ, Montecalvo A (2009). In situ-targeting of dendritic cells with donor-derived apoptotic cells restrains indirect allorecognition and ameliorates allograft vasculopathy. PLoS One.

[47] Xu DL, Liu Y, Tan JM (2004). Marked prolongation of murine cardiac allograft survival using recipient immature dendritic cells loaded with donor-derived apoptotic cells. Scand J Immunol.

[48] Albert ML, Sauter B, Bhardwaj N (1998). Dendritic cells acquire antigen from apoptotic cells and induce class I-restricted CTLs. Nature.

[49] Elliott MR, Ravichandran KS (2010). Clearance of apoptotic cells: implications in health and disease. J Cell Biol.

[50] Keating SE, Baran M, Bowie AG (2011). Cytosolic DNA sensors regulating type I interferon induction. Trends Immunol.

[51] Li Q, Xu B, Michie SA (2008). Interferon-alpha initiates type 1 diabetes in nonobese diabetic mice. Proc Natl Acad Sci USA.

[52] Bishop AE, Polak JM, Pickup J, Williams G (1997). The anatomy, organisation and ultrastructure of the islets of Langerhans. Textbook of diabetes.

[53] Roep BO, Tree TI (2014). Immune modulation in humans: implications for type 1 diabetes mellitus. Nat Rev Endocrinol.

[54] Thomas HE, McKenzie MD, Angstetra E, Campbell PD, Kay TW (2009). Beta cell apoptosis in diabetes. Apoptosis.

[55] Ryan A, Murphy M, Godson C, Hickey FB (2009). Diabetes mellitus and apoptosis: inflammatory cells. Apoptosis.

[56] Vargas F, Vives-Pi M, Somoza N (1998). Endotoxin contamination may be responsible for the unexplained failure of human pancreatic islet transplantation. Transplantation.

[57] Delovitch TL, Singh B (1997). The nonobese diabetic mouse as a model of autoimmune diabetes: immune dysregulation gets the NOD. Immunity.

[58] Trudeau JD, Dutz JP, Arany E (2000). Neonatal beta-cell apoptosis: a trigger for autoimmune diabetes?. Diabetes.

[59] Kassem SA, Ariel I, Thornton PS, Scheimberg I, Glaser B (2000). Beta-cell proliferation and apoptosis in the developing normal human pancreas and in hyperinsulinism of infancy. Diabetes.

[60] Mathis D, Vence L, Benoist C (2001). Beta-Cell death during progression to diabetes. Nature.

[61] Eizirik DL, Grieco FA (2012). On the immense variety and complexity of circumstances conditioning pancreatic beta-cell apoptosis in type 1 diabetes. Diabetes.

[62] Roggli E, Britan A, Gattesco S (2010). Involvement of microRNAs in the cytotoxic effects exerted by proinflammatory cytokines on pancreatic beta-cells. Diabetes.

[63] Han J, Zhong J, Wei W (2008). Extracellular high-mobility group box 1 acts as an innate immune mediator to enhance autoimmune progression and diabetes onset in NOD mice. Diabetes.

[64] Foulis AK, Liddle CN, Farquharson MA, Richmond JA, Weir RS (1986). The histopathology of the pancreas in type 1 (insulin-dependent) diabetes mellitus: a 25-year review of deaths in patients under 20 years of age in the United Kingdom. Diabetologia.

[65] Planas R, Carrillo J, Sanchez A (2010). Gene expression profiles for the human pancreas and purified islets in type 1 diabetes: new findings at clinical onset and in long-standing diabetes. Clin Exp Immunol.

[66] Imagawa A, Hanafusa T, Tamura S (2001). Pancreatic biopsy as a procedure for detecting in situ autoimmune phenomena in type 1 diabetes: close correlation between serological markers and histological evidence of cellular autoimmunity. Diabetes.

[67] Moriwaki M, Itoh N, Miyagawa J (1999). Fas and Fas ligand expression in inflamed islets in pancreas sections of patients with recent-onset type I diabetes mellitus. Diabetologia.

[68] Maas K, Chan S, Parker J (2002). Cutting edge: molecular portrait of human autoimmune disease. J Immunol.

[69] O’Brien BA, Geng X, Orteu CH (2006). A deficiency in the in vivo clearance of apoptotic cells is a feature of the NOD mouse. J Autoimmun.

[70] Green DR, Ferguson T, Zitvogel L, Kroemer G (2009). Immunogenic and tolerogenic cell death. Nat Rev Immunol.

[71] Marin-Gallen S, Clemente-Casares X, Planas R (2010). Dendritic cells pulsed with antigen-specific apoptotic bodies prevent experimental type 1 diabetes. Clin Exp Immunol.

[72] Planas R, Pujol-Borrell R, Vives-Pi M (2010). Global gene expression changes in type 1 diabetes: insights into autoimmune response in the target organ and in the periphery. Immunol Lett.

[73] Fadok VA, Bratton DL, Konowal A (1998). Macrophages that have ingested apoptotic cells in vitro inhibit proinflammatory cytokine production through autocrine/paracrine mechanisms involving TGF-beta, PGE2, and PAF. J Clin Invest.

[74] Phipps RP, Stein SH, Roper RL (1991). A new view of prostaglandin E regulation of the immune response. Immunol Today.

[75] Takano M, Nishimura H, Kimura Y (1998). Prostaglandin E2 protects against liver injury after Escherichia coli infection but hampers the resolution of the infection in mice. J Immunol.

[76] Izuora KE, Chase HP, Jackson WE (2005). Inflammatory markers and diabetic retinopathy in type 1 diabetes. Diabetes Care.

[77] Mangano K, Fagone P, Di Mauro M (2012). The immunobiology of apotransferrin in type 1 diabetes. Clin Exp Immunol.

[78] Valle A, Giamporcaro GM, Scavini M (2013). Reduction of circulating neutrophils precedes and accompanies type 1 diabetes. Diabetes.

[79] Diana J, Lehuen A (2014). Macrophages and beta-cells are responsible for CXCR2-mediated neutrophil infiltration of the pancreas during autoimmune diabetes. EMBO Mol Med.

[80] Diana J, Simoni Y, Furio L (2013). Crosstalk between neutrophils, B-1a cells and plasmacytoid dendritic cells initiates autoimmune diabetes. Nat Med.

[81] Barrett JC, Clayton DG, Concannon P (2009). Genome-wide association study and meta-analysis find that over 40 loci affect risk of type 1 diabetes. Nat Genet.

[82] Lee SC, Pervaiz S (2007). Apoptosis in the pathophysiology of diabetes mellitus. Int J Biochem Cell Biol.

[83] Moore F, Colli ML, Cnop M (2009). PTPN2, a candidate gene for type 1 diabetes, modulates interferon-gamma-induced pancreatic beta-cell apoptosis. Diabetes.

[84] Santin I, Moore F, Colli ML (2011). PTPN2, a candidate gene for type 1 diabetes, modulates pancreatic beta-cell apoptosis via regulation of the BH3-only protein Bim. Diabetes.

[85] Floyel T, Brorsson C, Nielsen LB (2014). CTSH regulates beta-cell function and disease progression in newly diagnosed type 1 diabetes patients. Proc Natl Acad Sci USA.

[86] Marroqui L, Santin I, Dos Santos RS (2014). BACH2, a candidate risk gene for type 1 diabetes, regulates apoptosis in pancreatic beta-cells via JNK1 modulation and crosstalk with the candidate gene PTPN2. Diabetes.

[87] Nogueira TC, Paula FM, Villate O (2013). GLIS3, a susceptibility gene for type 1 and type 2 diabetes, modulates pancreatic beta cell apoptosis via regulation of a splice variant of the BH3-only protein Bim. PLoS Genet.

[88] Santin I, Eizirik DL (2013). Candidate genes for type 1 diabetes modulate pancreatic islet inflammation and beta-cell apoptosis. Diabetes Obes Metab.

[89] Eizirik DL, Mandrup-Poulsen T (2001). A choice of death–the signal-transduction of immune-mediated beta-cell apoptosis. Diabetologia.

[90] Perruche S, Saas P, Chen W (2009). Apoptotic cell-mediated suppression of streptococcal cell wall-induced arthritis is associated with alteration of macrophage function and local regulatory T-cell increase: a potential cell-based therapy?. Arthritis Res Ther.

[91] Huynh ML, Fadok VA, Henson PM (2002). Phosphatidylserine-dependent ingestion of apoptotic cells promotes TGF-beta1 secretion and the resolution of inflammation. J Clin Invest.

[92] Lee YJ, Moon C, Lee SH (2012). Apoptotic cell instillation after bleomycin attenuates lung injury through hepatocyte growth factor induction. Eur Respir J.

[93] Voll RE, Herrmann M, Roth EA (1997). Immunosuppressive effects of apoptotic cells. Nature.

[94] Hugues S, Mougneau E, Ferlin W (2002). Tolerance to islet antigens and prevention from diabetes induced by limited apoptosis of pancreatic beta cells. Immunity.

[95] Xia CQ, Peng R, Qiu Y (2007). Transfusion of apoptotic beta-cells induces immune tolerance to beta-cell antigens and prevents type 1 diabetes in NOD mice. Diabetes.

[96] Trucco M, Giannoukakis N (2007). Immunoregulatory dendritic cells to prevent and reverse new-onset Type 1 diabetes mellitus. Expert Opin Biol Ther.

[97] Giannoukakis N, Phillips B, Finegold D, Harnaha J, Trucco M (2011). Phase I (safety) study of autologous tolerogenic dendritic cells in type 1 diabetic patients. Diabetes Care.

